# Conserved Sequences from Dengue Virus Genomes Form Stable G-Quadruplexes

**DOI:** 10.1021/acsinfecdis.4c00615

**Published:** 2024-12-12

**Authors:** Jessica L. Siemer, Thao T. Le, Ananya Paul, David. W. Boykin, Margo A. Brinton, W. David Wilson, Markus W. Germann

**Affiliations:** † Department of Chemistry, Georgia State University; Atlanta, Georgia 30303, United States; ‡ Department of Biology, Georgia State University; Atlanta, Georgia 30303, United States

**Keywords:** dengue virus, G-quadruplex, orthoflavivirus, RNA, nucleic acid structure, antivirals

## Abstract

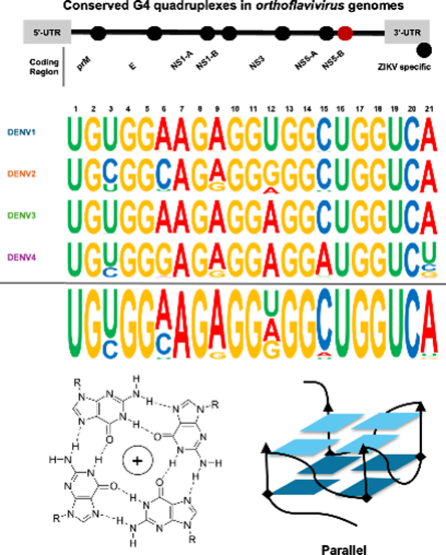

Arthropod-borne members of the genus *Orthoflavivirus* cause significant human disease. Four serotypes of dengue virus are endemic globally, and approximately 50 percent of the world’s population lives in a dengue-affected area. Complications from immunoenhancement occurring after a secondary infection with a different dengue serotype make vaccine development challenging. Antiviral therapies that target features conserved in all four serotypes would, therefore, be beneficial. Computational studies identified multiple potential G-quadruplex sites that are conserved in the RNA genome sequences of members of the genus *Orthoflavivirus*. Biophysical studies confirmed that the NS5-B quadruplex sequences obtained from viruses of each dengue serotype can form quadruplexes *in vitro*, and binding data showed that known quadruplex binders stabilized NS5-B quadruplexes for all four dengue serotypes.

Mosquito-borne diseases pose major threats to human health because of their ease of transmission and the expansion of endemic areas due to the increasing global temperatures. Mosquito-transmitted members of the genus *Orthoflavivirus*, such as dengue (DENV), West Nile (WNV), yellow fever (YFV), and Zika (ZIKV) viruses, cause significant human disease and morbidity worldwide. In 2023, the World Health Organization (WHO) reported over five million DENV cases in more than 80 countries.^1^ Despite half of the world’s population living in at-risk areas (Figure 1A), there are currently no antiviral treatments for DENV infection. To date, only three vaccines for DENV have reached Phase III clinical trials or have been licensed. However, the use of these vaccines in children and people naïve to dengue infection has been limited due to safety precautions. The complex pathology resulting from antibody-dependent enhancement after a sequential infection with a different serotype is a major challenge for developing a tetravalent DENV vaccine. For a vaccine to provide protection from severe diseases, such as dengue hemorrhagic fever and dengue toxic shock syndrome, neutralizing antibodies must be produced for all four serotypes, and this has proven to be difficult. Moreover, vaccination has the potential to worsen disease outcomes if a person is infected with a serotype for which the vaccine has not induced adequate neutralizing antibody levels. Therefore, the most widely adopted management strategies for DENV currently consist of vector control and population surveillance.

**Figure 1:**
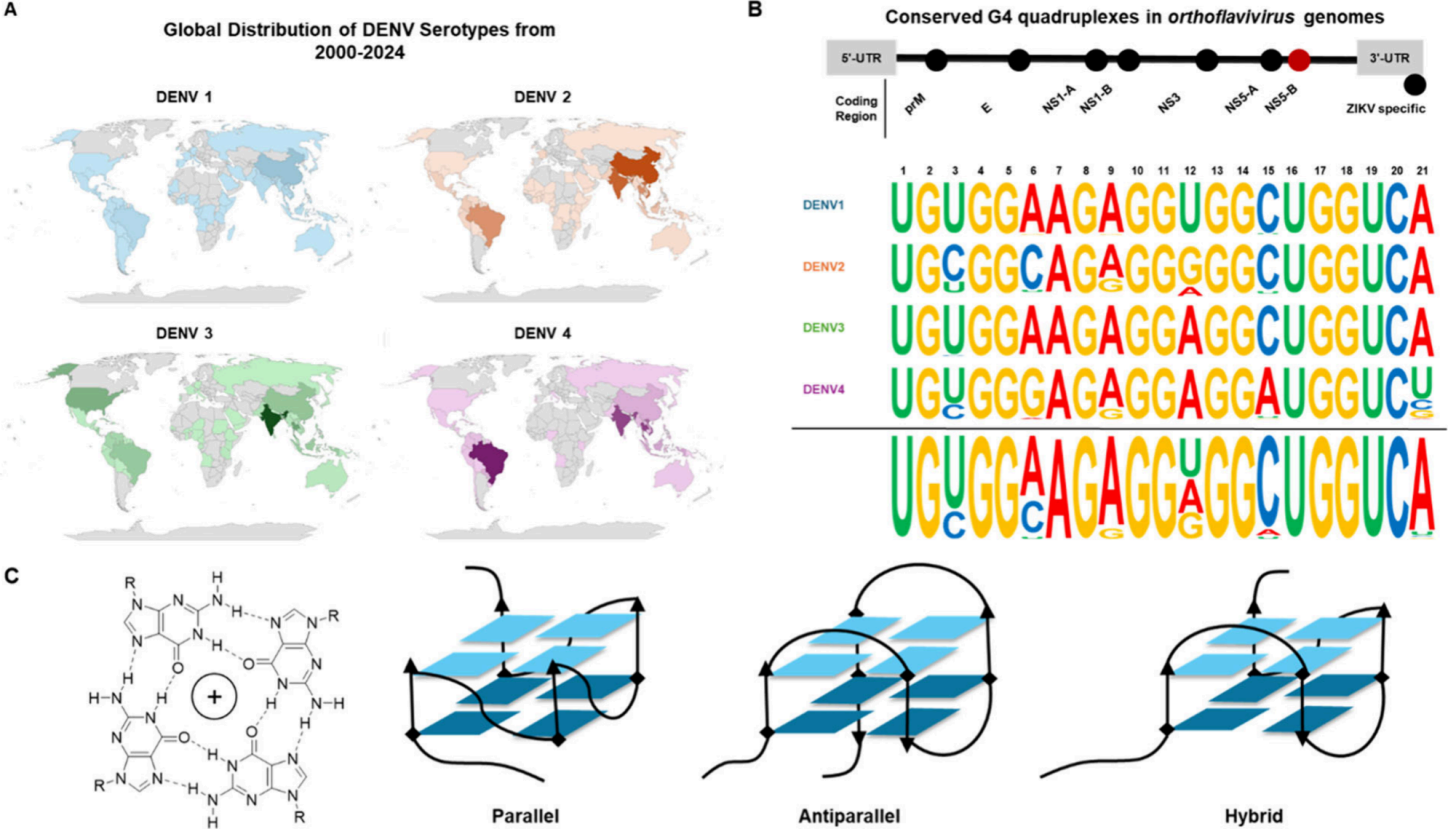
Global Prevalence of DENV Serotypes, Predicted NS5-B Quadruplex DENV Serotype Sequences, and Quadruplex Topology. (A) Global distribution of sequenced DENV serotype isolates from 2000 to 2024. Genome sequences (*n* = 35820) were mapped by serotype and geographic location. Darker colors indicate a higher frequency of deposited genomes relative to each serotype. (B) Consensus of each of the dengue serotype NS5-B quadruplex sequences. Seven conserved potential quadruplex sequences have been predicted in *Orthoflavivirus* genomes. The NS5-B sequence is highly conserved within each dengue serotype. The positions of the guanines predicted to form the tetrads are completely conserved across serotypes (Supplementary Table 1). (C) Tetrad structure and quadruplex topologies. Quadruplex tetrads form when four guanines form bonds with Hoogsteen symmetry. Loop conformations define the overall topology classification for a quadruplex.

The development of small-molecule therapeutics against DENV may be particularly advantageous, as it would directly target viral replication rather than rely on the activation of the host immune response. Antiviral therapies commonly target viral enzymatic proteins such as viral proteases and polymerases or viral protein interactions.^2,3^ However, to date, these strategies have not been broadly efficacious for DENV in part due to the genetic variability between serotypes. Therefore, alternative viral targets should be explored.

Computational studies have identified seven conserved potential G-quadruplex sequences in the genomes of >56 members of the genus *Orthoflavivirus* (Figure 1B).^4,5^ G-Quadruplexes form in guanine-rich DNA or RNA when two or more sets of four guanines bond in Hoogsteen geometry, forming tetrads that are further stabilized by a monovalent cation, such as potassium. The tetrads then stack forming a quadruplex structure which can vary in topology as dictated by their loop structures (Figure 1C).

While the functions of G-quadruplexes in orthoflavivirus genomes remain unknown, the genus-wide conservation of genomic sequences that can form these structures suggests that they may have functional importance. Host protein interactions with orthoflavivirus genomic quadruplexes have been implicated as a possible proviral mechanism for disrupting cellular stress responses.^6,7^ Additionally, several ligands have been developed to target quadruplexes, including the commercially available compounds BRACO-19, berberine, and pyridostatin (PDS), and have been shown to have antiviral effects against ZIKV, DENV, and tick-borne encephalitis.^8−11^ Additional characterization of the quadruplex sites of the four DENV serotypes will aid further exploration of quadruplex binders as viable therapeutics for DENV.

## DENV Serotype Conservation of the NS5-B Sequence.

 The NS5-B quadruplex sequence, which is located in the genome region encoding the methyltransferase domain of the viral polymerase, was selected for this study based on previous biophysical characterization of sequences from ZIKV and the crystal structure obtained for a structure derived from the WNV NS5-B sequence.^4,12^

The National Center for Biotechnology Information (NCBI) Virus portal was next used to retrieve 35,820 genomic DENV sequences which were deposited between January 2000 and June 2024 and had associated serotype and geographical information. These sequences were used to generate a global distribution map for each serotype (Figure 1A). From the total pool of deposited viral genomic sequences with serotype information, the 4,361 sequences that were isolated from humans and had less than 10 ambiguous bases were aligned and analyzed for nucleotide conservation in the NS5-B sequence (Figure 1B, Supplementary Table 2). The DENV 1 and 3 sequences each had the least sequence variability within their respective serotype. However, all four serotypes had a high degree of NS5-B sequence conservation with variations only occurring in the predicted quadruplex loop regions. While the NS5-B region is highly conserved, the variable nucleotides and the possible inaccessibility due to structure limit the use of potential therapeutics, such as peptide nucleic acids and Cas13-based RNA systems, for targeting all four serotypes with the same therapeutic since they rely on complementary sequences with a low tolerance for mismatches.^13,14^ Therefore, small molecules targeting RNA structures rather than precise sequences represent an attractive therapeutic strategy with broad serotype efficacy.

## All Serotype NS5-B Sequences Form Quadruplexes

**Figure 2:**
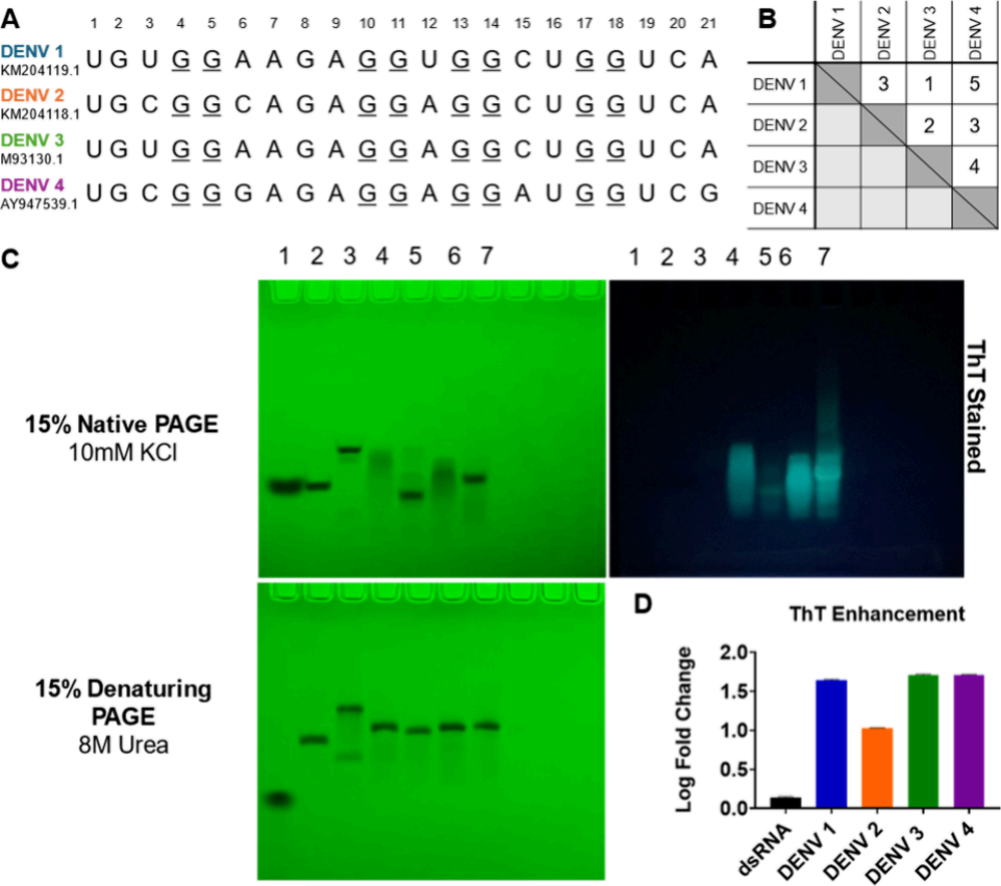
NS5-B RNAs from each of the four dengue serotypes form quadruplexes. (A) The NS5-B RNA sequences (21 nts) of DENV 1, DENV 2, DENV 3, and DENV 4 were analyzed. (B) Sequence difference matrix. (C) Denaturing and native PAGE of the DENV NS5-B quadruplex RNAs compared to nonquadruplex controls. Bands were detected by UV shadowing and ThT staining. Lane 1: Dye marker, Lane 2: 20-mer ssDNA T20, Lane 3: 20-mer dsRNA, Lane 4: DENV 1, Lane 5: DENV 2, Lane 6: DENV 3, Lane 7: DENV 4, (D) ThT fluorescence enhancement of the DENV NS5-B quadruplexes. Data are reported as the log fold change of the complex versus free ThT.

***In Vitro***. The 21-mer NS5-B sequences used in this study were selected from laboratory strains belonging to each DENV serotype (Figure 2A, Supplementary Table 1). The DENV 1 and DENV 3 NS5-B sequences have the highest degree of sequence similarity and differ in a single nucleotide (Figure 2B). DENV 2 and DENV 3 differ by two nucleotides. The largest number of nucleotide differences was observed for DENV 4 with five (DENV 1), three (DENV 2), and four (DENV 3) nucleotide differences.

The four DENV NS5-B 21-mers were analyzed by denaturing and native PAGE. Both the native and denaturing gels were visualized by UV shadowing, and the native gel was stained using the quadruplex-specific dye Thioflavin T (ThT) (Figure 2C). On the denaturing gel, each DENV RNA migrated to a similar distance as expected for single-stranded sequences of the same length. However, on the native gel, the bands of the DENV 1 and DENV 3 sequences were diffuse and migrated slower than the 20-mer ssDNA control, indicating greater conformational variability and the presence of less compact structures. In contrast, the DENV 2 and DENV 4 bands appear to be well-defined, indicating the presence of a major conformational species. The DENV 4 sequence had an electrophoretic mobility similar to that of the single-stranded T_20_ control, and the DENV 2 RNA migrated faster than the T_20_ ssDNA control suggesting that it forms a more compact structure. Despite the variability in electrophoretic mobility, each of the four DENV sequences was stained by ThT supporting the formation of quadruplexes. However, the fluorescence signal for DENV 2 NS5-B was less intense.

To further quantify ThT fluorescence enhancement by the DENV NS5-B RNAs, a cuvette-based assay was performed with ThT (Figure 2D). DENV 1, DENV 2, DENV 3, or DENV 4 NS5-B quadruplex RNA (2 μM) was added to 1 μM of ThT. After incubating for 10 min at 25 °C, the samples were excited at 412 nm, and fluorescence emission was measured at 485 nm. The NS5-B sequences for DENV 1, DENV 3, and DENV 4 significantly enhanced the ThT fluorescence. Lower enhancement was observed for the DENV 2 NS5-B RNA, which is consistent with the ThT-stained native PAGE result. In contrast, the addition of double-stranded RNA to ThT did not induce a significant fluorescence enhancement. A fluorescence titration experiment was performed by adding RNA to 1 μM ThT (Supplementary Figure 1A). Comparison of the ThT fluorescence intensities at a 14:1 molar ratio of RNA to ThT demonstrates that at saturation, the quantum yield of the ThT-DENV 2 NS5-B complex is over 3x lower than the other ThT-DENV NS5-B complexes (Supplementary Figure 1B). These data suggest that all four DENV sequences can form structures with significant π-stacking but that some structural differences exist between the DENV 2 NS5-B and the other DENV NS5-B sequences which prevent optimal fluorescence enhancement of ThT.

**Figure 3:**
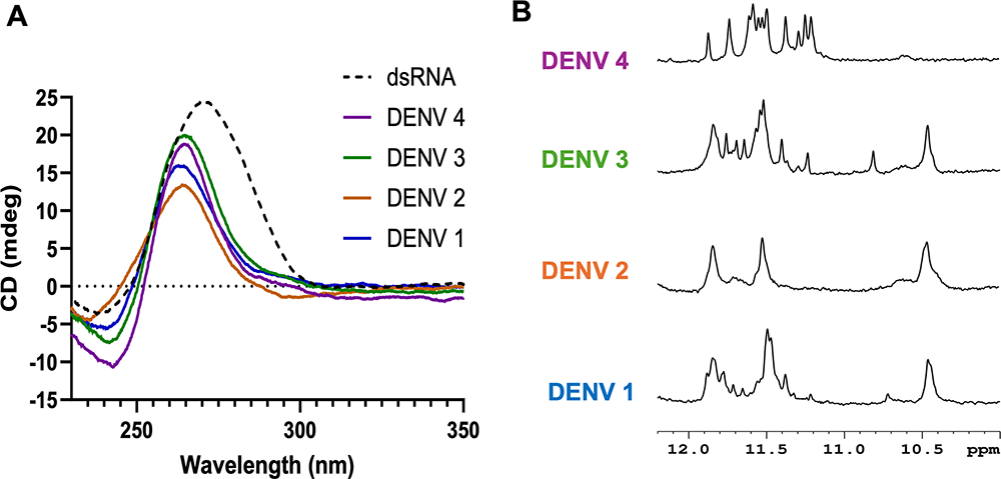
Spectroscopic characterization of DENV NS5-B sequences. (A) CD spectra of the DENV 1–4 NS5-B sequences (solid lines) and the 18-mer dsRNA control (dashed line). (B) ^1^H NMR spectra of the Hoogsteen resonances. NMR spectra were collected at 298 K using jump and return solvent suppression.

To further verify the formation of quadruplexes by DENV NS5-B sequences, CD spectra were collected for each sequence (Figure 3A). Signals characteristic of parallel-type structures with an *I*_*max*_ ≈ 264 nm and *I*_*min*_ ≈ 245 nm^15,16^ were observed for each of the DENV NS5-B sequences. Again, the spectra for the DENV 1, DENV 3, and DENV 4 NS5-B sequences were consistent, while the CD spectra of the DENV 2 NS5-B exhibited a broader signal. Additionally, the spectra for DENV 1 and DENV 2 have lower maximum intensities, with DENV 2 having the lowest maximal intensity. To confirm that DENV 1 forms a monomeric quadruplex, CD spectra were collected for DENV 1 NS5-B at 2 μM, 20 μM, and 100 μM (Supplementary Figure 2) in cuvettes with varying path lengths. The CD signals were the same for all three samples, which supports that the quadruplex formation is concentration-independent and therefore monomeric in that concentration range.

^1^H NMR spectra were collected for each of the DENV NS5-B sequences (Figure 3B). Imino proton signals were observed in the Hoogsteen region from 10 to 12 ppm for all four sequences (Figure 3B). NMR spectra of quadruplexes, particularly those of RNA G-quadruplexes, can vary significantly in appearance.^17,18^ The spectra in this region can range from a broad, featureless hump to distinct resonances for each of the imino protons involved in a Hoogsteen pairing.^4,19,20^ Broad proton signals are sometimes interpreted as evidence of multimeric species due to increased rotational correlation times. However, polymorphism and chemical exchange of monomeric quadruplexes can also cause these features.^12,19,21^ Even though the four RNA sequences studied contained the same placement, the number of GG elements of the NMR spectra varied significantly. The signals for DENV 2 NS5-B appeared broader and less intense, consistent with the data shown in Figure 2.

## Quadruplex Binders Stabilize DENV NS5-B.

**Figure 4:**
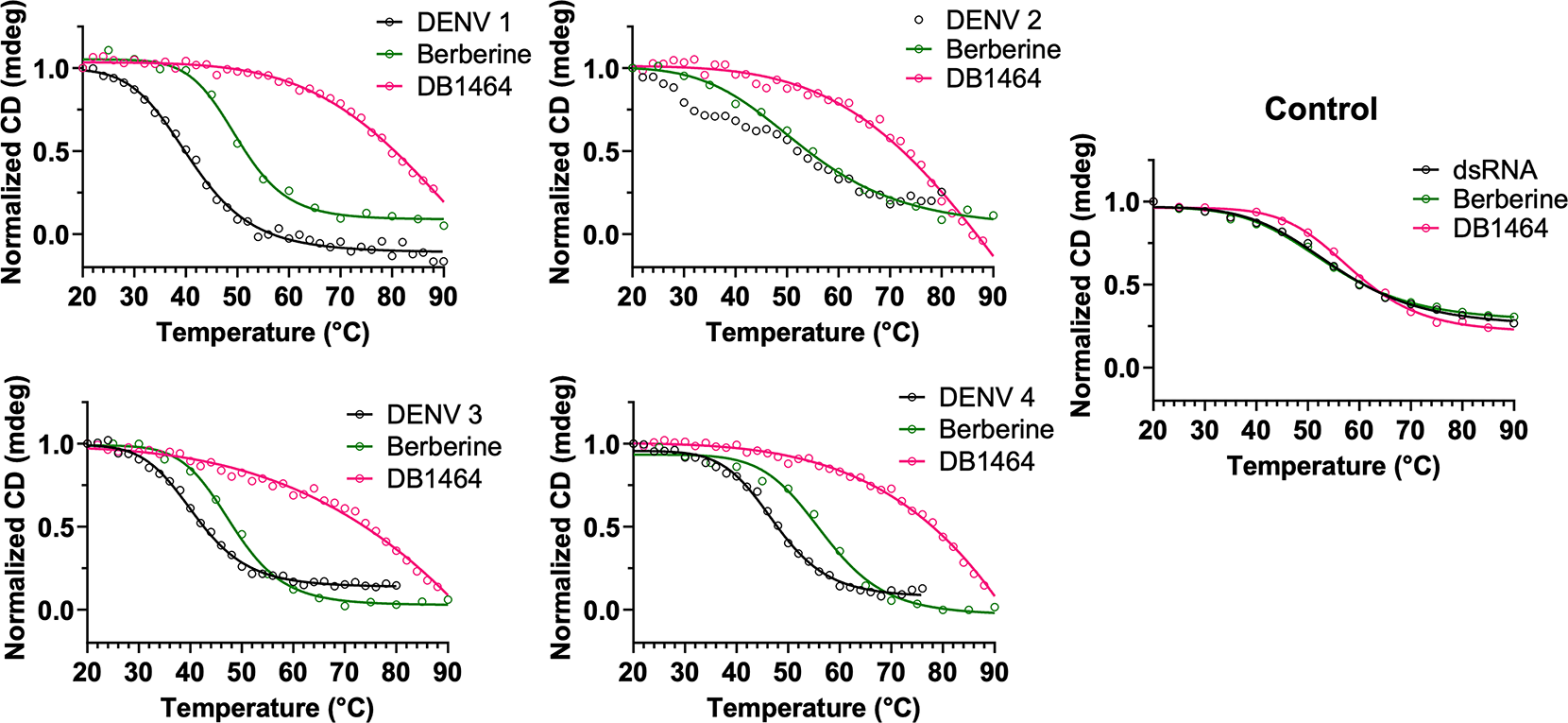
Quadruplex-specific ligands can bind to quadruplexes from all four DENV serotypes. CD melting curves for DENV NS5-B quadruplexes in the presence of berberine (green) and DB1464 (pink). An 18-mer hairpin was used as the dsRNA control.

 Examination of the quadruplex stability by CD thermal melting indicated that DENV 4 NS5-B forms the most stable quadruplex structure with a *T*_*m*_ of ∼48 °C, while both the DENV 1 and DENV 3 NS5-B RNAs melted at around 41 °C (Figure 4, Table 1). A biphasic melting curve was observed for DENV 2 NS5-B indicating a conformational rearrangement. DENV 1, DENV 3, and DENV 4 NS5-B did not show any significant hysteresis when comparing the CD melting curves upon heating or cooling. However, the DENV 2 melting curve has two transitions when heating but only one transition when cooling (Supplementary Figure 4). This observation, together with the data from previous experiments, provides insight into the structural differences between these quadruplexes. We hypothesize that the DENV 2 NS5-B sequence forms a less stable quadruplex that can undergo structural rearrangement, which broadens the CD and NMR signals and alters the π-stacking of the bases, resulting in decreased ThT fluorescence.

**Table 1:** Thermal stability of DENV NS5-B quadruplexes in the presence of quadruplex binding ligands

	*T*_*m*_ (°C)				
	DENV 1	DENV 2	DENV 3	DENV 4	dsRNA
**No ligand**	41.0 ± 0.2	N.D.^a^	41.0 ± 0.5	47.5 ± 0.7	55.1 ± 1.6
**Berberine**	50.1 ± 1.3	53.3 ± 2.2	48.1 ± 0.9	57.0 ± 1.9	54.1 ± 1.8
**DB1464**	>80.0	>80.0	>80.0	>80.0	58.6 ± 1.4

a The melting data for DENV 2 NS5-B was consistent with a multistep transition. The first transition did not have sufficient data points to fully define a biphasic function. Therefore, the *T*_m_ for the sequence was not able to be determined.

**Figure 5:**
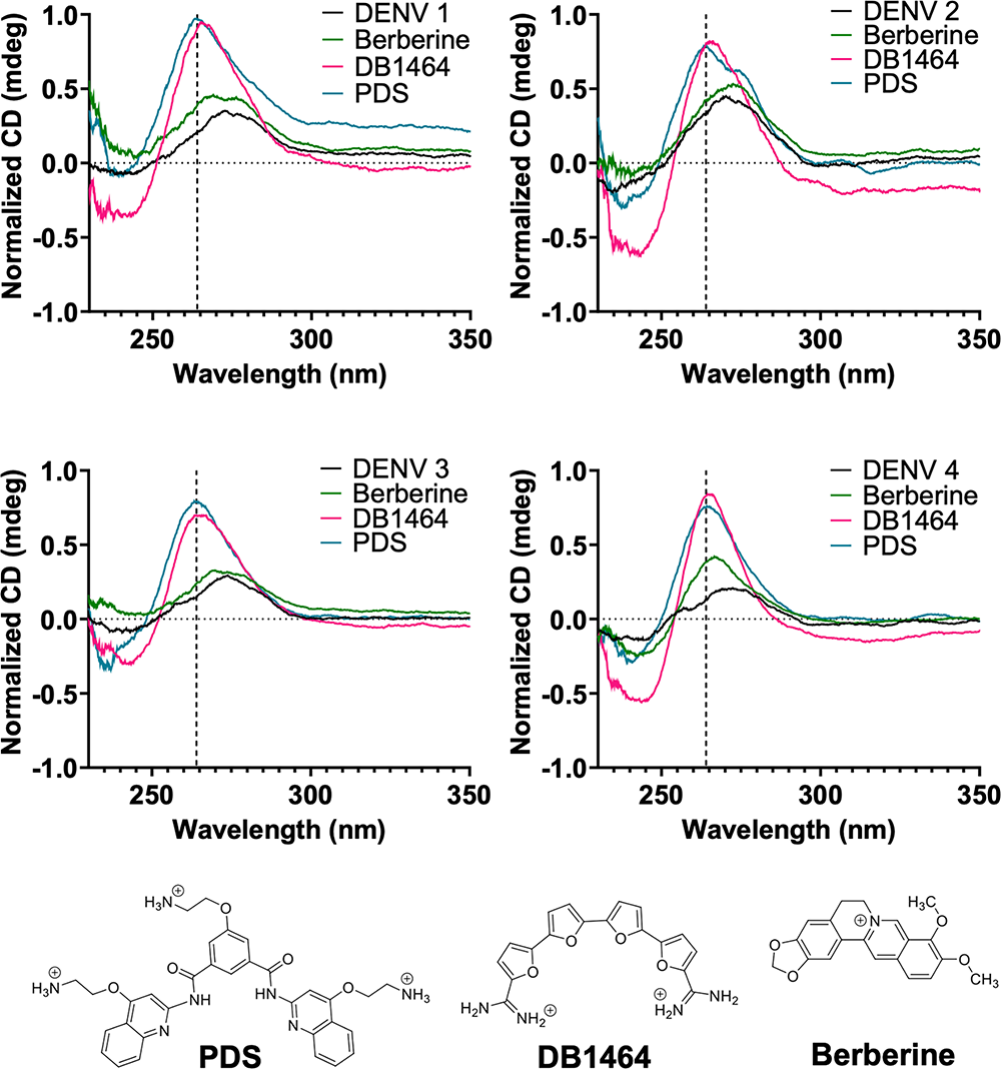
Quadruplex-specific ligands stabilize quadruplexes from all four serotypes. CD spectra from 230 to 350 nm of the DENV 1–4 NS5-B sequences were recorded at 60 °C with and without ligands. All ligands enhanced the 264 nm CD signal (vertical black dotted line) at 60 °C for each of the DENV quadruplex sequences.

Binding studies were conducted to explore the potential of using small molecules to target DENV quadruplexes. Two well-characterized commercially available quadruplex binders were selected for these studies: PDS, a strong quadruplex binder, and berberine, a weaker quadruplex binder. DB1464, a previously reported diamidine compound, was also selected based on its higher selectivity to quadruplexes relative to duplex DNA.^22−24^ At a 1:4 ligand/RNA ratio, all of the ligands stabilized the CD signal at 264 nm for each of the four DENV sequences at 60 °C (Figure 5). The data suggest that while the DENV 2 quadruplex appears susceptible to structural rearrangement, a quadruplex structure can still be stabilized. The CD *T*_*m*_ of each complex indicated that berberine was able to weakly stabilize three of the DENV NS5-B quadruplexes by 7–10 °C while DB1464 greatly enhanced the stability of the RNA quadruplexes to over 80 °C (Table 1) (Figure 5). PDS binding also yielded similar stabilization effects on the four DENV quadruplexes (Supplementary Figure 3). However, *T*_*m*_ was not able to be estimated, as it fell outside of the measurable range of the instrument.

In contrast, in the presence of a dsRNA control, berberine, PDS, and DB1464 displayed a minimal stabilization effect as indicated by a <4 °C change in *T*_*m*_ (Table 1, Supplementary Figure 5). This result not only confirmed the specificity of these quadruplex binding ligands to the DENV NS5-B quadruplexes over dsRNA but also demonstrated that targeting the quadruplex structure could be used as a potential method for therapeutic development against all four DENV serotypes.

## Conclusions.

 In summary, the bioinformatic analyses of the NS5-B quadruplex sequences from four different DENV serotype genome RNAs revealed a high level of conservation in the guanosine positions, but with some nucleotide variation occurring in the loop regions. This level of conservation is expected since this genomic region encodes the viral polymerase, NS5, and therefore must conserve both the structure and function of the encoded protein.^12^*In vitro* characterization of representative NS5-B sequences from each DENV serotype confirmed that all four sequences fold into quadruplex structures, with DENV 4 NS5-B having the highest thermal stability. Bioinformatic analyses have shown that GG motifs occur more frequently than longer G runs in viral RNA genomes.^7^ However, the stability of two-tetrad quadruplexes in the context of biological systems remains unclear. Given that all four sequences form monomeric quadruplexes combined with the structural insights from the WNV NS5-B crystal structure,^12^ which showed the importance of additional stabilizing features, it is likely that the DENV NS5-B sequences also form two tetrad quadruplexes by using noncanonical stabilizing structures.

Binding studies of the NS5-B quadruplexes with berberine, a weak quadruplex binder, and PDS, a strong quadruplex binder, demonstrated that both commercially available compounds can bind and stabilize the quadruplex sequences of all four DENV serotypes. Strong stabilization of the quadruplex sequences was also observed in binding experiments with DB1464, which was previously reported to have high selectivity and affinity toward quadruplexes.^20,21^ These data are complementary to published studies (Table 2) that have shown antiviral effects for several known quadruplex binding ligands against orthoflaviviruses. In particular, multiple studies have shown that berberine has antiviral effects against multiple orthoflaviviruses including DENV.^8−10,25^ These DENV studies hypothesized that berberine can act at the level of viral particle formation. Together with the binding data presented in this study, these results demonstrate the potential of targeting orthoflavivirus genomic quadruplexes as antiviral targets.

**Table 2:** Antiviral effects of known quadruplex binding ligands against orthoflaviviruses

Orthoflavivirus	Compound	Antiviral effect
TBEV^8^	PDS, cPDS, NMM, PhenDC3, Berberine	Suppression of viral E protein expression
ZIKV^11,25,26^	BRACO-19, TMPyP4, PDS	Reduction of genome replication and viral protein production
	Berberine	Reduction of viral RNA and infectious particles
DENV 1-4^9,10^	Berberine	Inhibition of infectious viral particle formation
WNV^13^	γPNA	Undetermined

Nevertheless, a major challenge in the development of quadruplex binders as therapeutics is achieving adequate specificity to limit off-target effects due to the presence of host cell quadruplexes. This study also establishes that minor changes in sequence can have significant effects on the overall structure of the quadruplex, presenting an additional challenge. Therefore, for a single therapeutic to effectively target all four DENV serotypes, a balance must be achieved between selectivity for the viral genomic quadruplexes over host quadruplexes while conserving recognition of multiple viral quadruplex conformations. Achieving this goal will be challenging. However, gaining more insight into the structure and function of these viral genomic quadruplexes is an essential initial step.

## Methods

### Serotype Mapping

The NCBI Virus database was queried to identify serotyped dengue genome sequences deposited between January 1, 2000, and June 6, 2024 (n = 35820). Results were further reduced to only include sequences that included geographical data. Serotype geographic frequency maps were generated by using the map chart function in Microsoft Excel 365.

### Sequence Consensus Determination

The NCBI Virus database was used to extract complete genome sequences that were isolated from human hosts for each serotype (DENV 1 n = 1727, DENV 2 n = 1453, DENV 3 n= 955, DENV 4 n = 226). The web-based alignment tool was used to analyze batches of 500 or fewer sequences. Nucleotide frequencies for the NS5-B sequences were compiled, and logos were generated for the sequence by converting the nucleotide frequency to scaled height for each nucleotide symbol in the logo.

### Oligonucleotides

RNA oligomers were obtained from Millipore and Sigma. The RNA was resuspended in RNase-free diH_2_O. For each experiment, stock RNA was diluted into the respective buffer to the appropriate concentration and heated to 95 °C for 5 min. The samples were cooled and subsequently stored at 4 °C overnight.

### Gel Electrophoresis

The RNA sequences were diluted to a final concentration of 25 μM concentration in 10 mM KCl, heated for 5 min at 95 °C, and stored at 4 °C overnight. The RNA samples were then run on separate 15% 19:1 polyacrylamide gels made with 1X TBE with 8 M urea (denaturing) or 10 mM KCl (native). The 1X TBE running buffer was also supplied with 10 mM KCl for the native condition. Bromophenol blue was used as a tracking dye. Images were captured using UV shadowing and staining with 10 μM Thioflavin T (Sigma-Aldrich).

### Fluorescence Enhancement Assay

Fluorescence enhancement assays were performed on a Cary Eclipse 300 Fluorescence Spectrophotometer. RNA and ThT were prepared in buffer containing 10 mM Tris-HCl, 50 mM KCl, and 1 mM EDTA at pH 7.5. For each sample, 2 μM of DENV 1, DENV 2, DENV 3, DENV 4 NS5-B quadruplex RNA, or double-stranded RNA control were added to 1 μM of ThT. After a 10 min incubation at 25 °C, the samples were excited at 412 nm and fluorescence emission was measured at 485 nm. The bar graph of ThT fluorescence enhancement for each DENV NS5-B quadruplex was generated as the log of the ratio of ThT-RNA fluorescence intensity to free ThT fluorescence intensity.

Fluorescence titration assays were performed on a PerkinElmer LS 55 Luminescence Spectrometer. RNA and ThT were prepared similarly as described above. For each sample 2–18 μM of DENV 1, DENV 2, DENV 3, DENV 4 NS5-B quadruplex RNA, or double-stranded RNA control was added to 1 μM of ThT. After each addition, the samples were incubated for 5 min, and the fluorescence emission scans were recorded at room temperature. Fluorescence graphs were created by using GraphPad 10.1.2.

### Circular Dichroism (CD) Spectroscopy

For CD melting or annealing experiments, spectra were collected on a JASCO 1500 CD spectrometer for the DENV 1, DENV 2, DENV 3, and DENV 4 NS5-B RNAs and double-stranded RNA control. Samples were prepared at 4 μM in 10 mM Tris-HCl, 50 mM KCl, and 1 mM EDTA at pH 7.5. PDS and berberine were purchased from MilliporeSigma. DB1464 was synthesized by Dr. Boykin as previously described.^20^ RNA samples containing berberine, PDS, or DB1464 were prepared at a 1:4 ratio, incubated at 80 °C for 5 min, and cooled overnight at room temperature.

For concentration-dependent CD analysis of DENV 1, RNA samples were diluted using sample buffer as described above. Spectra of DENV 1 at 100 μM, 20 μM, and 2 μM were collected at 25 °C using a 0.02, 0.1, and 1 cm cuvette, respectively.

The CD melting spectra were recorded across a 220 to 400 nm wavelength range, and the thermal melting was monitored at 264 nm corresponding to the parallel G-quadruplex topology or at 270 nm for the double-stranded RNA control. Spectra were recorded from 20 to 90 °C at a rate of 1 °C/min by using an integration time of 4 s. Annealing spectra were recorded similarly from 90 to 20 °C. CD data was normalized relative to the maximum CD signal at 264 nm. CD melting curves were generated by using a four-parameter logistic regression. CD Spectra and the thermal melting plot were created by using GraphPad 10.1.2.

### NMR

^1^H NMR spectrum was collected on a Bruker Avance 600 MHz NMR equipped with a 5 mm QXI probe. RNA samples were diluted with NMR sample buffer containing 10 mM Tris-HCl, 50 mM KCl, 1 mM EDTA, and 10% D_2_O to achieve a final concentration of 100 μM. Samples were adjusted to pH 7.5. 1D ^1^H imino spectra were acquired by using 1–1 jump-return solvent suppression.

## Supporting Information Available

Supplementary Table 1 Nucleotide Frequency for NS5-B Quadruplex Region in DENV Human Isolates; Supplementary Table 2 Oligonucleotide Sequences Used in Biophysical Characterization; Supplementary Figure 1 Fluorescence Titration of ThT with DENV NS5-B RNA; Supplementary Figure 2 Concentration Independence of DENV 1 NS5-B CD Signal; Supplementary Figure 3 CD Thermal Melting Spectra of DENV NS5-B Quadruplex Sequences; Supplementary Figure 4 CD Melting Curves of DENV NS5-B Sequences; Supplementary Figure 5 The Quadruplex Binders, Berberine, DB1464 and PDS with a dsRNA Control (PDF)
DENV isolates used in map and logo generation (DENV_consensus_and_map_source_datat.xlsx) (XLSX)
