# Supplementary material for: Conserved Sequences from Dengue Virus Genomes Form Stable G-Quadruplexes
Source: ACS Infect Dis. 2024 Dec 12;11(1):88–94. doi: 10.1021/acsinfecdis.4c00615 (PMC11731295; doi:10.1021/acsinfecdis.4c00615)
Supplement: Supplementary file 1 — id4c00615_si_001.pdf [file id4c00615_si_001.pdf]

Supporting Information

Conserved Sequences from Dengue Virus Genomes Form Stable G-Quadruplexes

Jessica L. Siemer[1*†], Thao T. Le[1†], Ananya Paul[1], David. W. Boykin[1], Margo A. Brinton[2], W. David Wilson[1], Markus W. Germann[1,2*]

[†]These authors contributed equally to this work

[1]Department of Chemistry, Georgia State University; Atlanta, GA 30303, USA.

[2]Department of Biology, Georgia State University; Atlanta, GA 30303, USA.

*Email: jsiemer1@gsu.edu; mwg@gsu.edu

Supplementary Figures

Supplementary Table 1. Nucleotide Frequency for NS5-B Quadruplex Region in DENV Human Isolates.

Position	1	2	3	4	5	6	7	8	9	10	11	12	13	14	15	16	17	18	19	20	21
**DENV 1** n=1727	**U**	**G**	**U**	**G**	**G**	**A**	**A**	**G**	**A**	**G**	**G**	**U**	**G**	**G**	**C**	**U**	**G**	**G**	**U**	**C**	**A**
**%**	100	100	99.54	100	100	98.38	100	100	98.96	100	99.94	99.65	100	99.83	97.22	100	99.94	99.94	99.88	99.94	99.88
			**C**			**G**			**G**		**U**	**C**		**C**	**U**		**U**	**U**	**C**	**U**	**G**
			0.4			1.62			1.04		0.06	0.17		0.17	2.78		0.06	0.06	0.12	0.06	0.12
			**G**									**A**									
			0.06									0.12									
												**G**									
												0.06									
**DENV 2** n=1453	**U**	**G**	**Y**	**G**	**G**	**C**	**A**	**G**	**A**	**G**	**G**	**G**	**G**	**G**	**C**	**U**	**G**	**G**	**U**	**C**	**A**
**%**	100	100		100	99.86	93.66	100	100	71.78	100	100	80.11	100	100	94.7	100	100	100	100	100	99.86
			**C**		**C**	**U**			**G**			**A**			**U**						**U**
			76.39		0.07	6.13			28.22			19.61			4.89						0.14
			**U**		**A**	**A**						**U**			**A**						
			23.61		0.07	0.07						0.28			0.41						
						**G**															
						0.14															
**DENV 3** n=955	**U**	**G**	**U**	**G**	**G**	**A**	**A**	**G**	**A**	**G**	**G**	**A**	**G**	**G**	**C**	**U**	**G**	**G**	**U**	**C**	**A**
**%**	100	100	99.27	100	100	100	99.9	99.9	99.9	100	100	99.58	100	100	99.48	100	100	100	100	100	99.9
			**C**				**C**	**A**	**U**			**G**			**U**						**C**
			0.73				0.1	0.1	0.1			0.21			0.52						0.1
												**U**									
												0.21									
**DENV 4** n=226	**U**	**G**	**U**	**G**	**G**	**G**	**A**	**G**	**A**	**G**	**G**	**A**	**G**	**G**	**A**	**U**	**G**	**G**	**U**	**C**	**Y**
**%**	100	100	71.68	100	100	96.46	100	100	79.65	100	100	99.56	100	100	95.13	100	100	100	100	100	
			**C**			**A**			**G**			**G**			**U**						**U**
			28.32			3.1			20.35			0.44			3.1						63.27
						**C**									**G**						**C**
						0.44									1.33						19.03
															**C**						**G**
															0.44						15.93
																					**A**
																					1.77
**Overall** n=4361	**U**	**G**	**U**	**G**	**G**	**A**	**A**	**G**	**A**	**G**	**G**	**U**	**G**	**G**	**C**	**U**	**G**	**G**	**U**	**C**	**A**
%	100.00	100.00	72.73	100.00	99.95	60.39	99.98	99.98	89.00	100.00	99.98	39.18	100.00	99.93	90.82	100.00	99.98	99.98	99.95	99.98	93.74
			**C**		**C**	**C**	**C**	**A**	**G**		**U**	**A**		**C**	**A**		**U**	**U**	**C**	**U**	**U**
			27.25		0.02	31.51	0.02	0.02	10.98		0.02	34.30		0.07	6.09		0.02	0.02	0.05	0.02	4.00
			**G**		**A**	**U**			**U**			**G**			**U**						**C**
			0.02		0.02	2.02			0.02			26.49			2.89						1.21
						**G**						**C**			**G**						**G**
						0.68						0.07			0.08						1.04

Supplementary Table 2. Oligonucleotide Sequences Used in Biophysical Characterization.

	Name of sequence	Sequence	Length
Controls	T_{20}	5'-TTT TTT TTT TTT TTT TTT TT-3'	20
	dsRNA (gels)	5'-CUG ACG AAG GCC UUC GUC AG-3'	20
	dsRNA (spectroscopy)	5'-AGA UCU UCC CCA GGA AUC U-3'	18
DENV G4s	DENV 1	5'-UGU GGA AGA GGU GGC UGG UCA-3'	21
	DENV 2	5'-UGC GGC AGA GGA GGC UGG UCA -3'	21
	DENV 3	5'-UGU GGA AGA GGA GGC UGG UCA -3'	21
	DENV 4	5'-UGC GGG AGA GGA GGA UGG UCG -3'	21

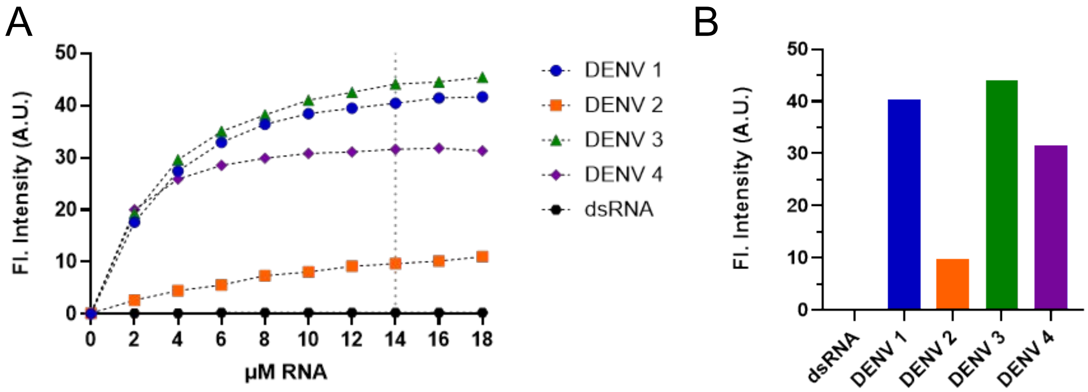

Supplementary Figure 1. Fluorescence Titration of ThT with DENV NS5-B RNA. A) 1 µM ThT was titrated with 2-18 µM of RNA to achieve saturation. B) Comparison of ThT fluorescence intensity for each sequence at a 14:1 RNA to ThT ratio.

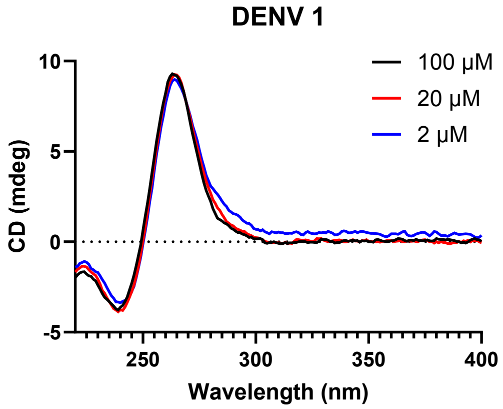

Supplementary Figure 2. Concentration Independence of the DENV 1 NS5-B CD Signal. (Blue) CD measurement of 2 µM DENV 1 NS5-B in a 1 cm cuvette, (Red) CD measurement of 20 µM DENV 1 NS5-B in a 0.1 cm cuvette, (Black) CD measurement of 100 µM DENV 1 NS5-B in a 0.02 cm cuvette

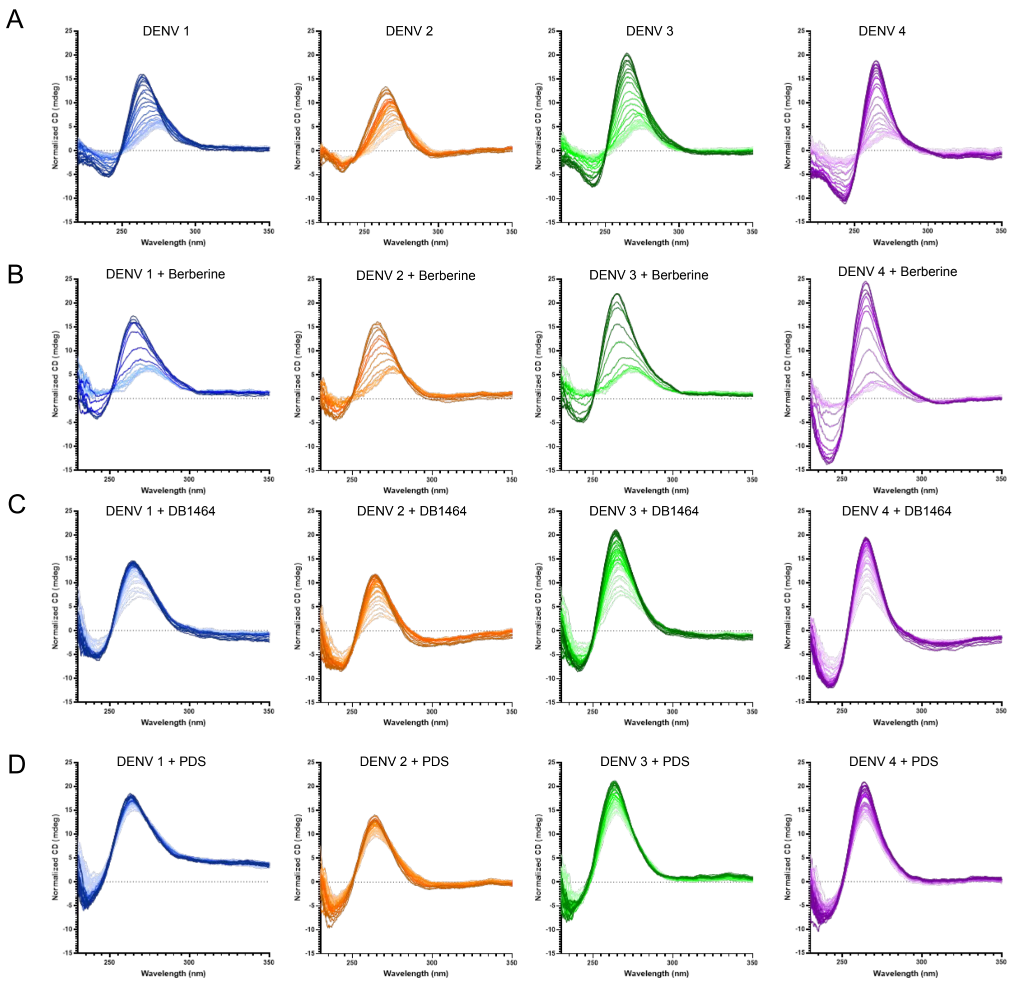

Supplementary Figure 3. CD Thermal Melting Spectra of DENV NS5-B Quadruplex Sequences. Spectra were collected from 220 to 350 nm at temperatures ranging from 20 °C to 90 °C. Compounds were added at a 4:1 ratio of ligand to RNA. A) CD melting spectra for each DENV NS5-B alone. CD melting spectra for each DENV NS5-B sequence bound to B) berberine C) DB1464 or D) PDS.

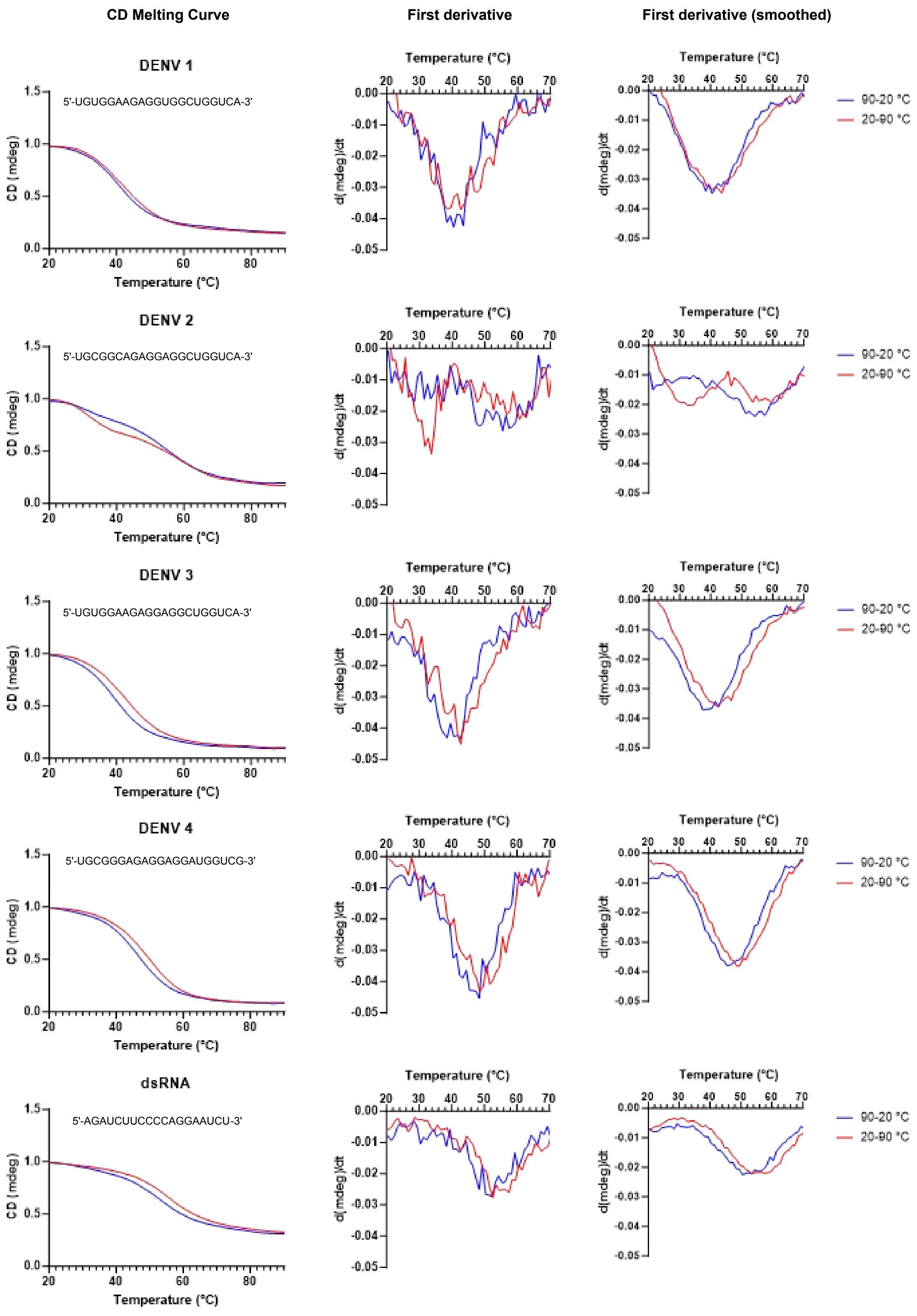

Supplementary Figure 4. CD Melting Curves of DENV NS5-B Sequences. (Red) Melting curve trace when heating from 20°C to 90°C (Blue) Melting curve trace when cooling from 90°C to 20°C. First derivatives of the melting curves with smoothing by averaging 5 nearest neighbors (left) or 15 nearest neighbors (right).

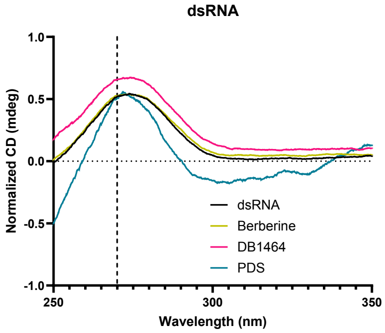

Supplementary Figure 5. The Quadruplex Binders, Berberine, DB1464 and PDS with a dsRNA Control. The CD spectra from 250 to 350 nm of an 18-mer hairpin dsRNA control with and without ligand at 60 °C. DB1464 weakly stabilizes the 270 nm CD signal for the dsRNA (vertical black dotted line) at 60 °C. However, berberine and PDS do not stabilize the dsRNA control at 60 °C. Aggregation was observed for the PDS sample which limited the CD measurement at lower wavelengths.
